# A randomized double-blind trial of intranasal dexmedetomidine versus intranasal esketamine for procedural sedation and analgesia in young children

**DOI:** 10.1186/s13049-024-01190-5

**Published:** 2024-03-04

**Authors:** Anna Nikula, Stefan Lundeberg, Malin Ryd Rinder, Mitja Lääperi, Katri Sandholm, Maaret Castrén, Lisa Kurland

**Affiliations:** 1 Department of Emergency Medicine and Services, Helsinki University, P.O. Box 4, 00014 Helsinki, Finland; 2 Department of Pediatric Anesthesia and Intensive Care, Astrid Lindgren Children’s Hospital, Karolinska University Hospital, 171 76 Stockholm, Sweden; 3 Department of Physiology and Pharmacology, Karolinska Institutet, 171 76 Stockholm, Sweden; 4 Department of Emergency Care for Children, Astrid Lindgren Children’s Hospital, Karolinska University Hospital, 171 76 Stockholm, Sweden; 5 Lääperi Statistical Consulting, Espoo, Finland; 6 Department of Emergency Medicine and Services, Helsinki University and Helsinki University Hospital, P.O. Box 4, 00014 Helsinki, Finland; 7 Department of Medical Sciences, Örebro University, 701 82 Örebro, Sweden; 8 Department of Emergency Medicine, Örebro University Hospital, Örebro, Sweden

**Keywords:** Intranasal, Dexmedetomidine, Esketamine, Procedure, Sedation, Analgesia, Children

## Abstract

**Background:**

Procedural sedation and analgesia are commonly used in the Emergency Departments. Despite this common need, there is still a lack of options for adequate and safe analgesia and sedation in children. The objective of this study was to evaluate whether intranasal dexmedetomidine could provide more effective analgesia and sedation during a procedure than intranasal esketamine.

**Methods:**

This was a double-blind equally randomized (1:1) superiority trial of 30 children aged 1–3 years presenting to the Emergency Department with a laceration or a burn and requiring procedural sedation and analgesia. Patients were randomized to receive 2.0 mcg/kg intranasal dexmedetomidine or 1.0 mg/kg intranasal esketamine.

The primary outcome measure was highest pain (assessed using Face, Legs, Activity, Cry, Consolability scale (FLACC)) during the procedure. Secondary outcomes were sedation depth, parents’ satisfaction, and physician’s assessment.

Comparisons were done using Mann–Whitney U test (continuous variables) and Fisher’s test (categorical variables).

**Results:**

Adequate analgesia and sedation were reached in 28/30 patients. The estimated sample size was not reached due to changes in treatment of minor injuries and logistical reasons. The median (IQR) of highest FLACC was 1 (0–3) with intranasal dexmedetomidine and 5 (2–6.75) with intranasal esketamine, (*p*-value 0.09). 85.7% of the parents with children treated with intranasal dexmedetomidine were “very satisfied” with the procedure and sedation compared to the 46.2% of those with intranasal esketamine, (*p*-value 0.1). No severe adverse events were reported during this trial.

**Conclusions:**

This study was underpowered and did not show any difference between intranasal dexmedetomidine and intranasal esketamine for procedural sedation and analgesia in young children. However, the results support that intranasal dexmedetomidine could provide effective analgesia and sedation during procedures in young children aged 1–3 years with minor injuries.

*Trial registration*: Eudra-CT 2017-00057-40, April 20, 2017. https://eudract.ema.europa.eu/

**Supplementary Information:**

The online version contains supplementary material available at 10.1186/s13049-024-01190-5.

## Background

The need for pain management and sedation are common in the Emergency Departments (EDs) treating pediatric trauma patients. Every fifth pediatric ED visit is due to trauma [1] and a peak incidence is seen in young children [1, 2]. Their injuries are often classified as minor [3] and can be treated in the ED. As these procedures can be painful and frightening for a child, adequate sedation and analgesia need to be ensured. In addition, the absence of effective procedural sedation and analgesia (PSA) can result in a negative experience for both children and their parents, which in turn can impact future procedures or even hospital visits [4, 5]. Despite the awareness, children still fail to receive adequate PSA due to lack of knowledge and evidence on different possibilities for PSA as reported in recent surveys from Scandinavia and Canada [6, 7].

There are several ways to administer drugs for PSA. Intravenous (IV) administration is widely used. A common risk with IV drugs is deeper sedation than intended, which motivates a sound knowledge of pharmacological effects [8]. The lack of this expertise prevents clinicians from using IV drugs. Another reason for considering alternative delivery routes is the need of an IV-line as cannulation can be difficult and stressful for the child [9]. Intranasal (IN) administration is a non-invasive and easy method and is therefore appealing.

IN esketamine (sKET), an S-enantiomer of ketamine, is routinely used for PSA in Astrid Lindgren Children’s hospital (ALB), but to ensure every child an adequate PSA other options are also needed.

IN dexmedetomidine (DEX) is an interesting alternative as it rarely has clinically significant effects on respiratory or cardiovascular systems [10–17] and causes minimal discomfort when administered [18]. IN DEX has successfully been used as a sedative for non-painful procedures e.g., for imaging [12, 14, 19, 20]. It has been shown to have both a good analgesic effect during IV-cannulation [17] and sedative effect during dental treatment [16]. However, there are limited results for IN DEX in PSA in the ED.

The objective of this study was to evaluate whether IN DEX could provide more effective analgesia and sedation during a painful procedure than IN sKET among young children 1–3 years of age presenting to the ED with minor injuries.

## Methods

### Trial design

This prospective, equally randomized (1:1), double-blind, parallel group trial was conducted in a large pediatric ED. This study was monitored by an independent regulatory unit, Karolinska Trial Alliance. Ethical approval was obtained from the Regional Ethical Review Board Stockholm. And this study was registered with European Clinical Trial Registry. The trial protocol is presented as Additional file 1.

The CONSORT guidelines [21] were used for reporting our data.

### Participants

#### Inclusion and exclusion criteria

Children 1–3 years old who presented to the ED with a laceration in need of suturing or a burn covering less than 4% of the body surface area and required PSA were eligible for enrolment. Injury was assessed and the need for PSA determined by the ED physician (mainly physicians in training in pediatrics, emergency medicine or general medicine) according to local guidelines. The trial physician was contacted, and the inclusion and exclusion criteria were revised. Patients with American Society of Anesthesiologist physical status classification (ASA) [22] ≥ III, current respiratory tract infection, impaired level of consciousness or any other neurologic symptoms as well as hypersensitivity to the trial drugs were excluded. In addition, children of parents with insufficient understanding of the Swedish language could not be enrolled in this study as written information of the trial was provided in Swedish. Signed informed consent was given by the parents.

#### Setting and location

This study was conducted in the pediatric ED at Astrid Lindgren Children’s hospital (ALB), Karolinska University Hospital in Stockholm, Sweden. ALB ED has 50 500 annual visits and offers medical care to children and adolescents with all levels of trauma, injuries, and sickness.

### Outcomes and definitions

The primary outcome was pain, measured as the highest level of pain during the procedure. Procedure was defined for lacerations as suturing of the wound and for burns as wound debridement and dressing, local guidelines for the procedures were followed. Pain was assessed with Face, Legs, Activity, Cry, Consolability scale (FLACC) [23], which has been validated for procedural pain assessment by Nilsson et al. [24]. FLACC scores were classified as following: 0 = no pain, 1–3 = mild discomfort, 4–6 = moderate pain and 7–10 = severe pain [25, 26]. We considered a change of two points on the FLACC as clinically significant.

Secondary outcomes were sedation depth, parents’ satisfaction, and ED physician’s assessment of the feasibility of the procedure. Ramsay sedation scale [27] was used to evaluate sedation depth, as it is one of the most widely used tools for observationally based sedation assessment and it has been used in studies assessing sedation with intranasal dexmedetomidine [12, 14]. Ramsay score 1 = awake, 2 = awake; co-operative, orientated and tranquil, 3 = awake; responds to commands only 4 = asleep; reacts with a brisk response to a light glabellar tap or a loud auditory stimulus, 5 = asleep; reacts with a sluggish response to a light glabellar tap or a loud auditory stimulus, 6 = asleep; does not respond to pain. We considered a change of one point as clinically significant.

Parent/parents who were with the child from administration of the trial drug until recovery, received a questionnaire with four questions: (1) in your opinion; how much pain did your child have during the procedure on a scale of 0–10 (the revised Faces Pain Scale (FPS-R) [28] was shown to the parents), (2) what was your opinion about the sedation and the procedure on a scale of 1–5 (1 = not satisfied, 5 = very satisfied). (3) if your child needs procedural sedation again in the future, would you prefer the same management? (yes/no), (4) if not what would you wish to be different?

The ED physician graded the feasibility of performing the procedure on a scale of 1–5 (1 = very easy, 5 = very difficult).

### Interventions

All patients included in the study received oral paracetamol (40 mg/kg) no later than 1–1.5 h before the procedure.

Patients were enrolled and randomized before the administration of local anesthesia. Buffered lidocaine (10 ml 1% lidocaine + 2 ml NaHCO_3_ 6 M) was used for local anesthesia. The burn area was covered with lidocaine-soaked gauze for 20–30 min before the procedure. Wounds were infiltrated with buffered lidocaine for a minimum of 5 min prior to the procedure. The maximum lidocaine dose without adrenaline was 5 mg/kg and with adrenaline 7 mg/kg.

DEX 100 mcg/ml and sKET 25 mg/ml were used without dilution. A 1 ml syringe with a nasal atomizer was used for drug administration. 2.0 mcg/kg DEX and 1.0 mg/kg sKET were used following the local and national guidelines [29]. The dose was equally divided between both nostrils when the recommended volume per nostril (0.3 ml/nostril) [30] was exceeded.

Patients were monitored from the administration of the trial drug until the patient had recovered, and Ramsay score was 1*.* Pain (FLACC score) and sedation (Ramsay score) were assessed at least every 5 min before and during the procedure and every 10 min after the procedure. Oxygen saturation (SpO_2_) and heart rate (HR) were monitored continuously. SpO_2_ and HR were recorded at the same timepoints with FLACC and Ramsay unless significant changes occurred at other times. Any aberration from normal values described in Pediatric Advanced Life Support (PALS) [31] were considered significant. Assessment and monitoring were done by two experienced pediatricians (AN, KS) who were familiar with both the FLACC and Ramsay scales. Five patients were initially evaluated together to ensure uniform assessment.

The procedure was started when Ramsay score 2 was reached. If it was not reached within 30 min and the child was not co-operative the procedure was not carried through within trial protocol and other sedation was used for the treatment. Patients were able to leave the ED when Ramsay score was 1 and the patient had returned to the habitual condition.

### Randomization and blinding

Patients were randomized to two groups: 1) IN DEX 2.0 mcg/kg, 2) IN sKET 1.0 mg/kg. We used block randomization (blocks of 10 subjects (5 from both arms), except one block with 12 subjects). A physician not participating in the trial created a randomization list after a random draw and filled the opaque envelopes with information (trial drug and dose table) and numbered them according to the list. Envelopes were then used in number order. Randomization was kept in a sealed envelope during trial period and the seal was intact at the time of trial closure. The blocks were used in order and the order was not known by the physician performing the assessment of the patient.

The trial physician, ED physician and nurses caring for the patient, as well as the patient and parents were blinded to the trial drug, as were all other staff working in the ED. Trial drug was prepared and administered by a nurse who was not involved in the care of the patient otherwise. Trial physician, ED physician and nurses caring for the patient were not present at the time of drug administration. Patients and their parents did not have knowledge about the trial drugs (e.g., smell or taste, nasal irritation) or the difference between the volume of the two drugs.

### Statistical methods

An a priori power analysis was conducted to test the null hypothesis of this superiority trial. We aimed for a mean difference of 2 on FLACC which would be the smallest clinically relevant difference. Assuming a within-group standard deviation of 2.5 we would need n = 26 patient per group to obtain 0.80 power for the test.

Continuous variables are presented using medians and interquartile ranges (IQRs) and tested with Mann–Whitney U test and tested correlations using the Spearman method. Categorical variables are presented using counts and percentages and tested using Fisher’s test. We considered p-values below 0.05 significant. All analyses were done using R version 4.2.2. [32].

## Results

Patient enrollment was conducted between July 2017 and October 2019 when one of the two trial physicians was present*.*

**Fig. 1:**
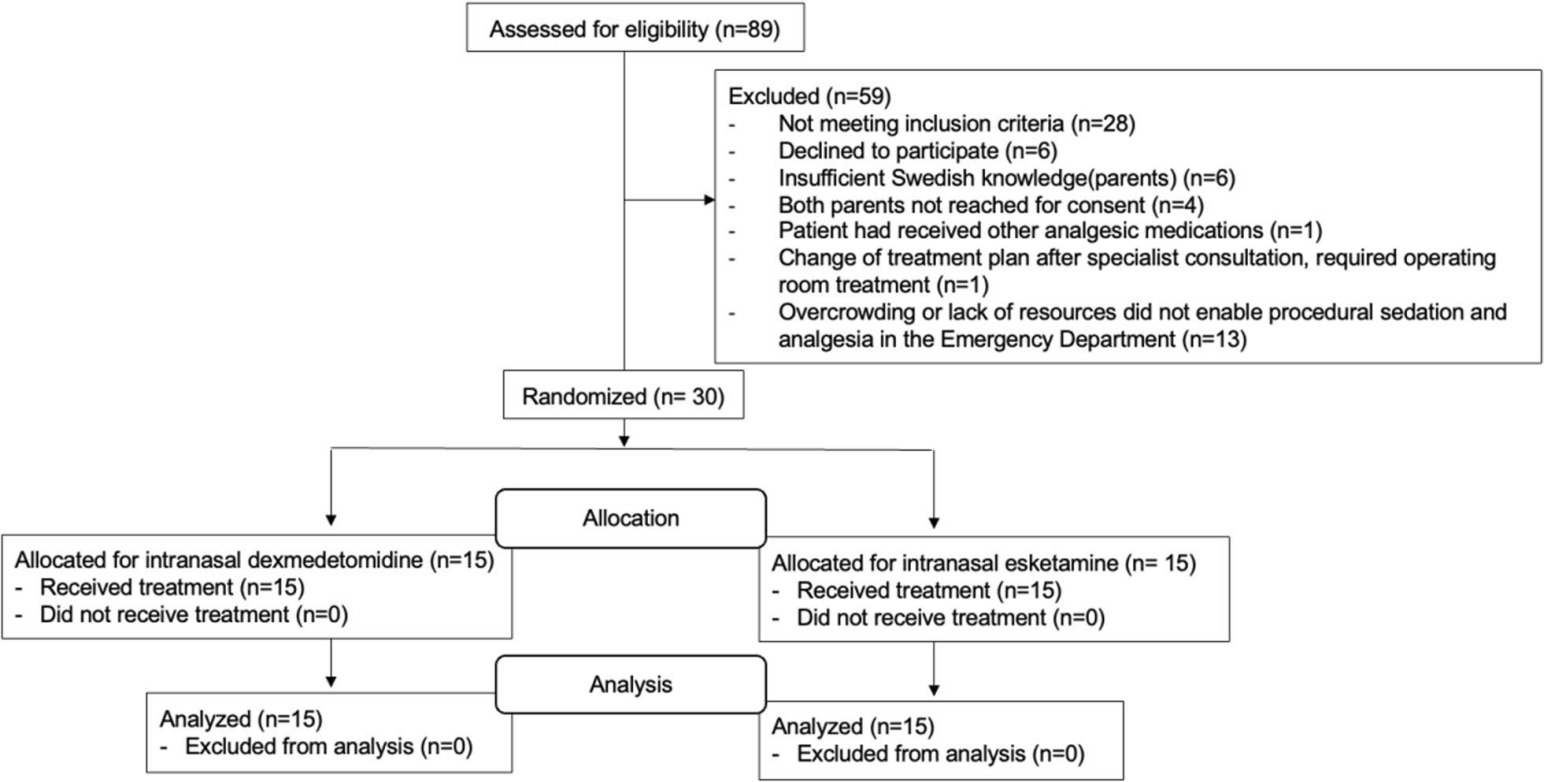
Participant flow chart

89 patients were assessed for eligibility and 30 patients were randomized, see flow chart (Fig. 1). The groups were similar regarding baseline demographics (Table 1). The estimated sample size was not reached due to changes in treatment of minor injuries and logistical reasons.

**Table 1:** Baseline demographics of the trial population

Demographic feature	All	Intranasal dexmedetomidine	Intranasal esketamine
Number of patients	30	15	15
Age (mo) mean	24	26	23
Weight (kg) mean	12,7	12,9	12,4
Male (%)	19 (63,3)	8 (53,3)	11 (73,3)
Female (%)	11 (36,7)	7 (46,7)	4 (26,7)
Laceration	27	12	15
Burn	3	3	0
Missing	0	0	0

Adequate analgesia and sedation to complete the procedure was reached in 28/30 patients. One patient in group IN sKET did not reach Ramsay 2 and was not co-operative, hence procedure could not be carried out within the trial protocol. With one patient in group IN DEX the procedure was started but due to high pain reactions (FLACC 9) despite of sedation level of Ramsay 2 the procedure could not be carried out within the trial protocol. The data for each group and comparisons between the groups are summarized in Table 2.

**Table 2:** Pairwise comparisons between the intranasal dexmedetomidine and intranasal esketamine groups

Variable	Levels	Intranasal dexmedetomidine N 15	Intranasal esketamine N 14	*P*-value	Missing
Higghest FLACC		1 (0–3)	5 (2–6.75)	0.09	
Highest Ramsay		3 (2–3)	2 (1–2)	0.02	
ED Phycisian grading	1 = Very easy	8 (57.1%)	3 (21.4%)	0.21	1 IN DEX
	2 = Easy	4 (28.6%)	6 (42.8%)		
	3 = Not easy—not difficult	2 (14.3%)	2 (14.3%)		
	4 = Difficult	0	2 (14.3%)		
	5 = Very difficult	0	1 (7.1%)		
Parental satisfaction	1 = Not satisfied	0	1 (7.7%)	0.10	2 IN DEX
	2 = Somewhat satisfied	0	0		
	3 = Neutral	0	1 (7.7%)		
	4 = Satisfied	2 (14.3%)	5 (38.4%)		
	5 = Very satisfied	12 (85.7%)	6 (46.2%)		
Pain assessed by parents (FPS-R)		2 (0–5)	5 (0–8)	0.44	2 IN DEX

Continuous variables are presented using medians and IQRs and tested with Mann–Whitney U test. Categorical variables are presented using counts and percentages and tested using Fisher’s test

*IN DEX* intranasal dexmedetomidine; *IN sKET* intranasal esketamine, *FPS-R* revised Faces Pain Scale

**Fig. 2:**
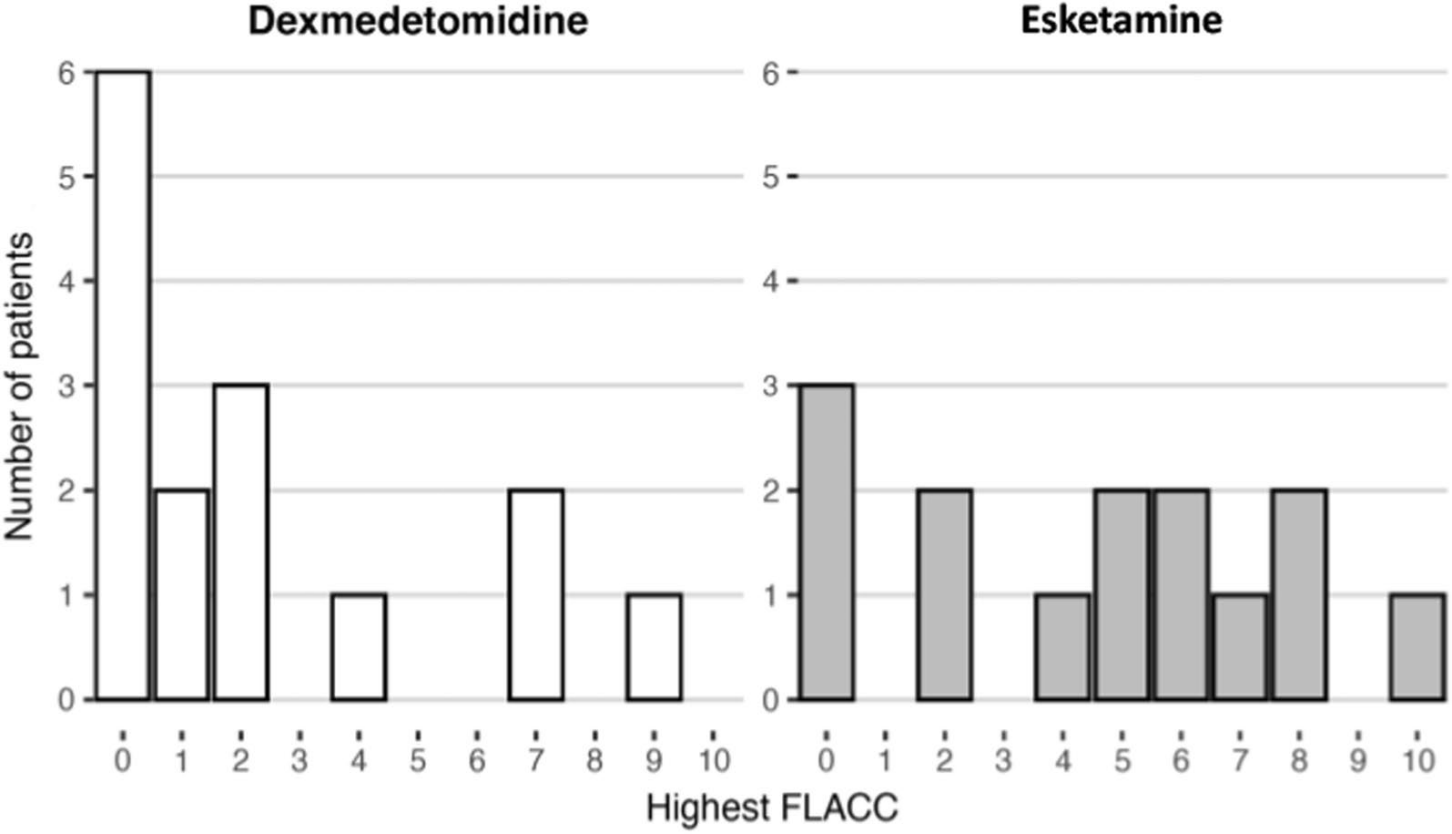
Highest pain level during the procedure. Pain level was assessed with Face, Legs, Activity, Cry, Consolability scale (FLACC). This diagram shows the distribution of FLACC scores per drug. The median (IQR) of highest FLACC with patients receiving intranasal dexmedetomidine was 1 (0–3) and in group intranasal esketamine 5 (2–6.75), *p*-value 0.09

FLACC on all patients before administration of trial drug was 0. The median (IQR) of highest FLACC was 1 (0–3) in group IN DEX and 5 (2–6.75) in group IN sKET, there was no statistical difference between the groups (p-value 0.09). Pain assessment as FLACC is shown in Fig. 2.

**Fig. 3:**
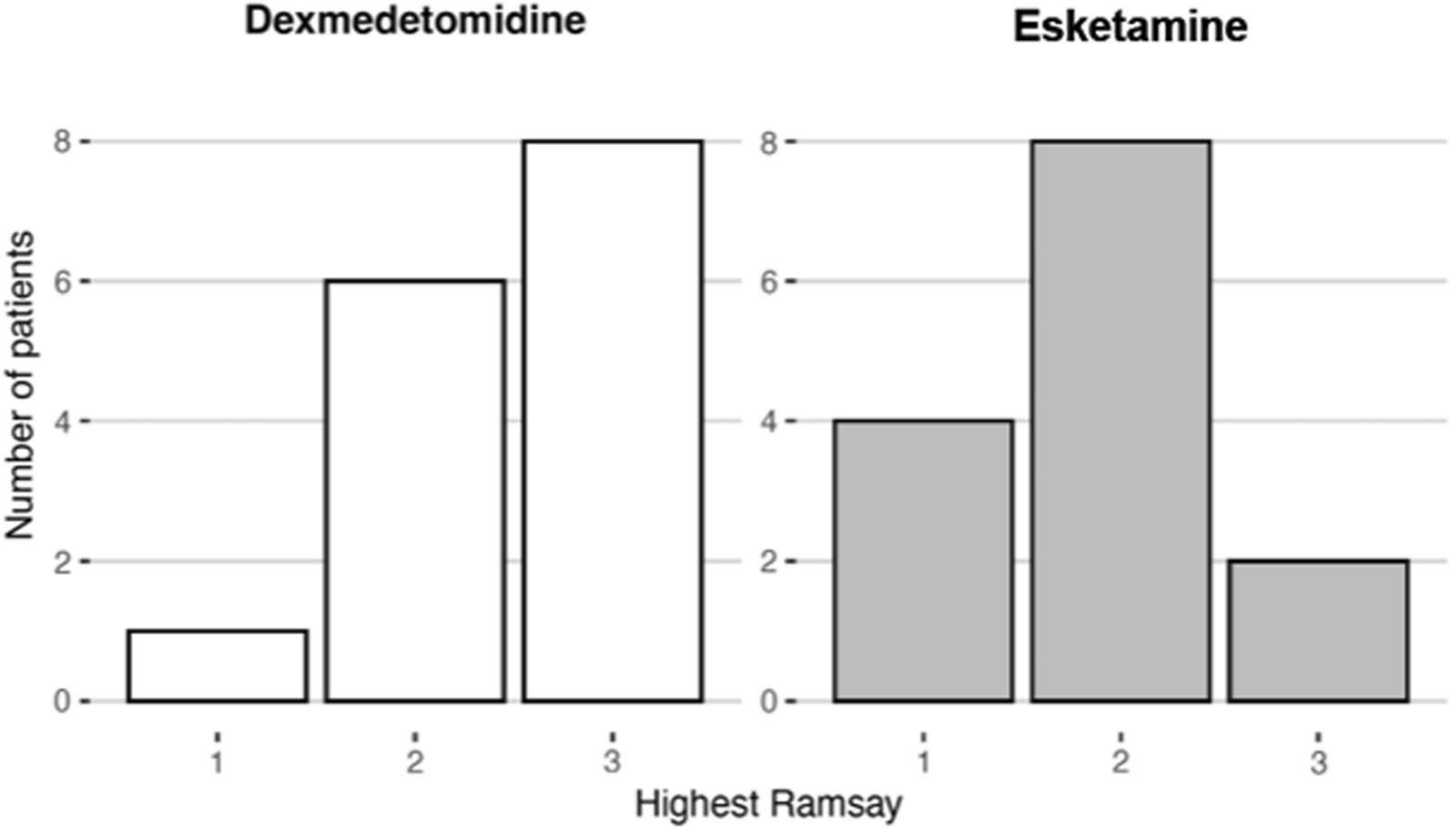
Highest sedation score during the procedure. Sedation level was assessed with Ramsay sedation scale. This diagram shows the distribution of Ramsay scores per drug. The median (IQR) of highest Ramsay with patients receiving intranasal dexmedetomidine was 3 (2–3) and in group intranasal esketamine 2 (1–2), *p*-value 0.02

The median (IQR) of Ramsay score was 3 (2–3) with IN DEX and 2 (1–2) with IN sKET, p-value 0.02. Sedation assessment as Ramsay is shown in Fig. 3.

### Parents’ opinion

One parent in group IN sKET did not wish to answer the questionnaire. The parents of the two patients not receiving sufficient sedation and analgesia with the trial drug only stated they did not wish for the same treatment in the future. 12 (85.7%) of the parents in the group IN DEX were”very satisfied” with the procedure and sedation and 6 (46.2%) in group IN sKET, p-value 0.10. 13/14 of the parents of patients receiving IN DEX and 10 out of 13 parents of patients receiving IN sKET would be satisfied with the same procedural sedation and analgesia if needed in the future. Parents’ assessment of their child’s pain score (FPS-R) median (IQR) was 2.5 (0–5) in group IN DEX and 5 (0–8) in group IN sKET, p-value 0.44.

### ED physician’s opinion

The ED physician graded the feasibility of the procedure as 1 = very easy in 8 (57.1%) cases in group IN DEX, whereas 3 (21.4%) cases in group IN sKET were graded 1, p-value 0.21.

### Adverse events

No severe adverse events were reported. Two patients in group IN sKET and one in group IN DEX had SpO2 94% very briefly, no drop in HR was noted and no intervening from hospital staff was required. No patients had HR under the 1st age normative percentile.

## Discussion

The results of the current study support that IN DEX can provide effective analgesia and sedation during PSA among young children aged 1–3 years with minor injuries, however, it could not be proven to be superior to IN sKET. This lack of significant difference could be explained by the low sample size as a result of not reaching estimated sample size rather than true lack of difference. The estimated sample size was not reached due to changes in treatment of minor injuries and logistical reasons, for details see Limitations below. More parents were satisfied with IN DEX as compared with IN sKET, and these parents would also prefer the same procedural sedation and graded their children’s pain as lower. More procedures were graded as very easy by ED physicians when IN DEX was used as compared with IN sKET.

In our study patients receiving IN DEX had generally lower pain scores during the procedure than those in IN sKET group. Three quarters of the patients receiving IN DEX had no pain or only mild discomfort whereas in the IN sKET group, the majority had moderate to severe pain during the procedure. Two studies [16, 17] have analyzed pain level during PSA, and their results support the findings in our study.

The lack of evidence may have prevented physicians from considering IN DEX as an option for PSA for painful procedures in the ED [7]. In addition, the lack of alternatives may have contributed to the use of physical restraints [6]. We believe that despite incomplete inclusion, and therefore not reaching calculated sample size, these results show clinically relevant information in relation to IN DEX analgesic and sedative effect during painful procedures in young children.

Deeper sedation (Ramsay ≥ 3) was reached more often with IN DEX than IN sKET. Deeper sedation typically has been shown to provide better circumstances for the procedure which, in turn, often contributes to better outcome. In our study ED physicians graded the procedure very easy to perform more often with patients who had received IN DEX than IN sKET. Neville et al. [33] showed the superiority of IN DEX to IN midazolam as an anxiolytic prior to laceration repair in young children. The sedative effect of IN DEX during non-painful procedures has been reported in previous studies [12, 14, 19, 20] and during dental treatment [16]. Results from these studies support the current results that IN DEX provides a deeper sedation and is safe to use.

Parents’ opinion about the PSA is important as their experience and satisfaction can affect a child’s anxiety and fear [34]. In our study, parents to children treated with IN DEX graded their general opinion of the management of the procedure and sedation as the category “very satisfied” more often than was the case in the IN sKET group, although statistical significance was not shown. Parents in group IN DEX also estimated their child’s pain during the procedure to be lower than in group IN sKET. These results are encouraging for the use of IN DEX for PSA in the ED for young children, although further study is required to confirm these results.

Besides providing safe and good PSA, good circumstances to perform a procedure is essential for an ED physician. In our study ED physicians graded the feasibility of performing the procedure very easy more often with patients receiving IN DEX than IN sKET.

We did not see any significant effects on the respiratory or cardiovascular systems in either patient group, but a conclusion of the safety of the drugs cannot be assessed with this small sample size. Adverse events of IN DEX are reported in many studies although no clinically significant changes on systolic blood pressure, HR, respiratory rate or SpO^2^ has been seen with the use of IN DEX [11–15, 35]. These results support that IN DEX is safe to use for PSA in the ED with adequate monitoring.

### Limitations

The main limitation of this study is not reaching the power needed to show statistical significance. We did not reach the estimated sample size during the study period of two years even though this study was conducted in a large pediatric ED. There are several explanations for this. During the study period there was a change in the streaming protocol for children with minor injuries in the region due to the opening of a number of low-acuity emergency care centers in Stockholm treating children with minor injuries which resulted in fewer patients attending the ALB ED, reducing the number of eligible patients for our study. Furthermore, the topical lidocaine, adrenaline, tetracaine gel and tissue glue changed the treatment practice for laceration repair and therefore procedural sedation was needed less frequently. Moreover, the study team could not be expanded as the Karolinska Trial alliance strongly recommended physicians to perform the patient assessment during the trial. Hence the study was required to be done by physicians, which in turn was a limiting factor as there are few experienced pediatric emergency physicians available both at ALB and other centers. Continued enrollment during 2020 was initially planned but could not be executed due to the Covid-19 pandemic. In addition, we could potentially have reached sample size if study drugs were administered in two doses, this would have allowed children weighing more than 15 kg to be enrolled.

There were two trial physicians doing all the assessments, which can be seen as both a strength and a limitation. A strength, as this reduces the variability in the FLACC and Ramsay scores. On the other hand, this obviously can have affected the number of patients enrolled as the trial physicians could not be present at all times.

The dosage of sedative drugs used in this study can be discussed. For IN sKET we followed the national guidelines from the Swedish Medical Product Agency regarding procedural sedation and analgesia for children [29]. The recommended dose for IN sKET is 1.0 mg/kg in children and with a maximum weight of 15 kg as otherwise the intranasal volume would become too large for good absorption from the nasal mucosa. 23 children were excluded from this study as they weighed more than 15 kg. This could have been avoided by administering the drugs twice with a specific time interval. However, we chose to follow the Swedish guidelines. Before 2017 most of the studies on IN DEX were done with the maximum dose of 2.0 mcg/kg [13, 33]. In addition, the local guidelines and the experience from IN DEX for imaging in ALB impacted our choice of the dose. In 2023 Poonai et al. suggested that 3 or 4 mcg/kg could be considered as an optimal dose of IN DEX in laceration repair [36].

### Generalisability

This trial was limited to the age group 1–3 years. Lacerations and burns are common minor injury types in this age group and were therefore selected for this trial. Unfortunately, all patients with burns received IN DEX for trial drug as a follow to the random allocation. These limitations need to be taken into consideration when treating children in other age groups and different procedures requiring analgesia and sedation are in question.

## Conclusion

This study was underpowered and did not show any difference between intranasal dexmedetomidine and intranasal esketamine for procedural sedation and analgesia in young children. However, the results support that intranasal dexmedetomidine could provide effective analgesia and sedation during procedures in young children aged 1–3 years with minor injuries.

## Supplementary Information

**Additional file 1.** Clinical trial protocol.

## Data Availability

The datasets used and analyzed during the current study are available from the corresponding author on reasonable request.

## Abbreviations

ALB: Astrid Lindgren Children’s Hospital
ASA: Anesthesiologist physical status classification
DEX: Dexmedetomidine
ED: Emergency Department
FLACC: Face, Legs, Activity, Cry, Consolability scale
FPS-R: Revised Faces Pain Scale
HR: Heart rate
IN: Intranasal
IQR: Interquartile range
IV: Intravenous
sKET: Esketamine
PALS: Pediatric Advanced Life Support
PSA: Procedural sedation and analgesia
SpO_2_: Oxygen saturation
